# Gastrointestinal symptoms, faecal short-chain fatty acids and dietary intake in older adults: the Hordaland Health Study 3

**DOI:** 10.1007/s00394-026-04077-6

**Published:** 2026-08-06

**Authors:** Tilde Martinsen, Caroline Jensen, Jan Gunnar Hatlebakk, Jørgen Valeur, Ottar Nygård, Gülen Arslan Lied

**Affiliations:** 1 Department of Clinical Medicine, Faculty of Medicine, Centre for Nutrition, University of Bergen, Bergen, Norway; 2 Section of Gastroenterology, Department of Clinical Medicine, Haukeland University Hospital, 5021 Bergen, Norway; 3 Department of Medicine, National Centre for Functional Gastrointestinal Disorders, Haukeland University Hospital, Bergen, Norway; 4 Unger-Vetlesen Institute, Lovisenberg Diaconal Hospital, Oslo, Norway; 5 Institute of Clinical Medicine, University of Oslo, Oslo, Norway; 6 Department of Clinical Science, University of Bergen, Bergen, Norway; 7 Department of Heart Disease, Haukeland University Hospital, Bergen, Norway

**Keywords:** Gastrointestinal symptoms, Irritable bowel syndrome, Older adults, Faecal short-chain fatty acids, Dietary intake

## Abstract

**Purpose:**

Ageing is associated with a decline in gastrointestinal (GI) functions, but data on GI symptoms in older adults remain limited. This study assessed the prevalence of GI symptoms, irritable bowel syndrome (IBS), and faecal short-chain fatty acids (SCFAs) in older adults, and associations between GI symptoms and faecal SCFAs, dietary intake, and BMI.

**Methods:**

907 older adults (66–69 years, 50.7% females) from the community-based Hordaland Health Study 3 − Microbiota study were included. GI symptoms and IBS status were assessed by validated questionnaires (Gastrointestinal Symptom Rating Scale, Rome IV-criteria, IBS-Severity Scoring System), dietary intake by a 279-item food frequency questionnaire, and the concentration of SCFAs were measured from faecal samples.

**Results:**

33.9% experienced at least one GI symptom, 16.4% reported IBS-like symptoms, and 5.0% had IBS according to Rome IV-criteria. Indigestion was the most common GI symptom (24.2%). Females had a higher total mean score of GI symptoms (*p* = 0.021), with higher frequencies of constipation (*p* = 0.006) and abdominal pain (*p* = 0.031), compared to males. Males had higher concentration of total faecal SCFAs (*p* = 0.004) and all major SCFAs compared to females. Total faecal SCFAs were negatively associated with constipation, independently of dietary fibre intake, total energy intake, sex and BMI (B =  − 0.006, 95% CI [− 0.009, − 0.004], *p* < 0.001). We observed a positive correlation between total faecal SCFA concentrations and diarrhoea (*r* = 0.072, *p* = 0.039) and looser stool consistency (*r* = 0.185, *p* < 0.001); however, these associations did not persist in the regression analyses. All GI symptoms positively correlated with increasing BMI.

**Conclusion:**

GI symptoms are common in older adults, and IBS is at least as prevalent as observed in the general adult population. Improving dietary habits and preventing overweight and obesity may reduce the risk of GI symptoms and improve gut health in older adults.

**Supplementary Information:**

The online version contains supplementary material available at https://doi.org/10.1007/s00394-026-04077-6.

## Introduction

The rapidly growing population of older adults (≥ 65 years old) puts major pressure on the healthcare system [1]. Older adults are at higher risk of chronic diseases, including gastrointestinal (GI) disorders [2, 3]. Maintaining a well-functioning gut has been recognized as an important part of overall health and quality of life in this age group [4]. The ageing process is associated with several alterations along the GI tract that may negatively affect its function and increase the risk of GI symptoms, such as abdominal pain and constipation [5, 6]. Emerging evidence also suggests that advancing age is associated with loss of beneficial gut microbes and gut-related metabolites, including short-chain fatty acids (SCFAs), resulting in dysbiosis of the gut microbiota [7]. Gut dysbiosis has recently been associated with impaired gut barrier integrity, which may result in harmful substances entering the bloodstream, potentially triggering systemic low-grade inflammation, known as “*inflammaging*” [8]. Inflammaging is characterized by elevated levels of pro-inflammatory markers suggested to contribute to an unhealthy ageing process [9].

There is a substantial variation in the prevalence of GI symptoms in community-dwelling older adults when comparing findings from different countries [10, 11]. The identification of GI symptoms in older adults also remains challenging. However, it is well recognized for its multifactorial aetiology, including low dietary fibre intake, lack of physical activity, and use of medications, as well as disturbances in swallowing function, reduced gastric emptying, and loss of colon motility [5]. Additionally, overweight, obesity and female gender have been associated with increased risk of GI symptoms [12]. It has also been demonstrated that alterations in the concentrations of SCFAs, produced by the gut microbiota, might influence GI health and increase the risk of disorders of gut-brain interaction, including irritable bowel syndrome (IBS) [13]. IBS is diagnosed based on symptom-based criteria (Rome criteria) and characterized by chronic relapsing abdominal pain associated with altered bowel habits, without any organic lesions [14]. The global prevalence ranges from 3.8–9.2% in the general population, depending on criteria used [15]. To date, few studies have assessed the prevalence of IBS in older adults [16], and to the best of our knowledge, no study have used the latest Rome criteria (Rome IV).

The association between dietary intake and GI symptoms is widely accepted, and more than two out of three individuals with IBS report postprandial exacerbation of GI symptoms after a meal [17]. Notably, dietary intake is one of the major factors that influence the levels of the SCFAs [18]. The SCFAs are suggested to promote the gut barrier function and to have anti-inflammatory effects, indicating that they are key players in maintaining GI homeostasis [19]. To date, we have limited knowledge about the concentrations of faecal SCFAs in older adults [20]. Identifying how faecal SCFAs, dietary factors, and BMI relate to GI symptoms and IBS could be a future target for preventive and therapeutic intervention to promote gut health in the older population [21].

The objective of the sub-study of the Hordaland Health Study 3 (HUSK3) was to assess the prevalence of GI symptoms, including IBS, as well as the concentration of faecal SCFAs in a large sample of community-dwelling adults > 65 years, and investigate the associations between GI symptoms and faecal SCFAs, dietary intake, and BMI.

## Methods

### Study population

In this study we have included data from the HUSK3—Part 2 Microbiota Study (https://husk.w.uib.no/husk3-mikrobiota/). This is a sub-study of HUSK3 (https://husk-en.w.uib.no/), a community-based population study conducted in Bergen, Norway, including men and women born 1950–51, living in Bergen and its surroundings, and who participated in the previous HUSK1 and HUSK2. The HUSK studies are a collaboration between the National Health Screening Service (now the Norwegian Institute of Public Health), the University of Bergen, and local health authorities. All participants in HUSK3 were invited to take part in HUSK3—Part 2 Microbiota Study. The study was conducted in accordance with the Declaration of Helsinki and approved by the Regional Committee for Medical and Health Research Ethics of Western Norway (2018/329/REC West). All subjects provided written informed consent.

### Data collection

**Fig. 1:**
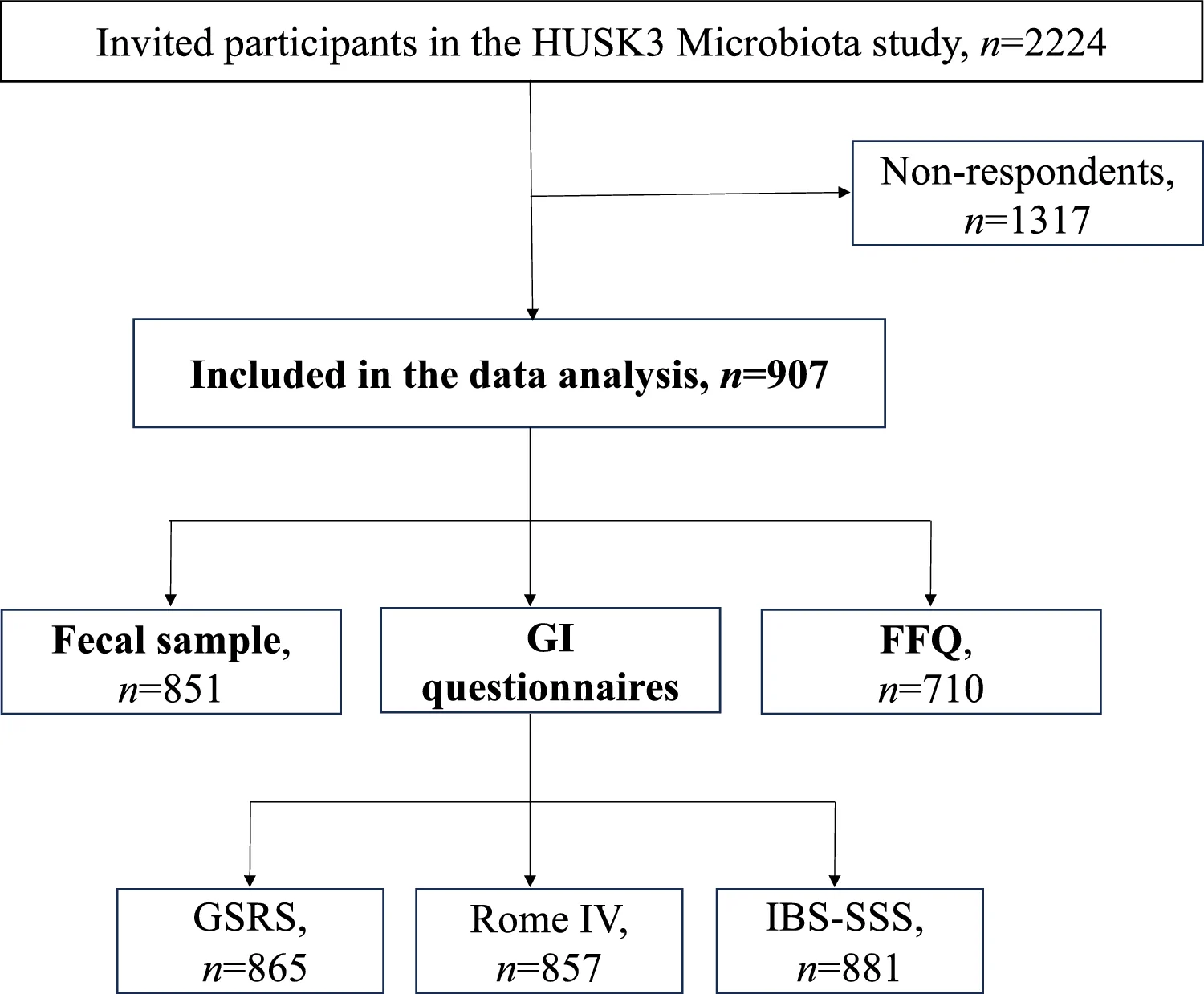
Flowchart of the inclusion process and the data collection in Hordaland Health 3 Study (HUSK3)—Microbiota Study. GI, gastrointestinal; FFQ, food frequency questionnaire; GSRS, gastrointestinal symptom rating scale; IBS-SSS, irritable bowel syndrome severity scoring system

Data was collected between September 2019 and July 2021. The participants in the HUSK3 study met for a study visit at the Research Unit for Health Surveys, University of Bergen, Bergen, Norway, where they underwent several health examinations, including anthropometric and clinical measurements, and dietary assessment. During this visit, the participants were invited to take part in the HUSK3—Part 2 Microbiota Study. Interested participants were telephoned by a research nurse and received information about the study. Those accepting to participate received a folder with written information about the study, a consent form, materials for the collection of a faecal sample and validated GI questionnaires, described in more detail in the “Gastrointestinal symptom questionnaires”–“Dietary assessment and anthropometric measurements” section. Figure 1 gives an overview of the inclusion process in the study.

### Gastrointestinal symptom questionnaires

The presence and severity of GI symptoms was assessed by Gastrointestinal Symptom Rating Scale (GSRS) [22]. Briefly, the GSRS consists of 15 questions divided into five categories: abdominal pain, reflux, indigestion, diarrhoea, and constipation, where each question refers to GI symptoms during “the past week”. The participants reported their symptoms on a scale ranging from 1 to 7. The higher the overall score, the more severe GI symptoms. We used a mean value of 3 or more as the cut-off for bothersome GI symptoms, in line with previous studies [11].

The Rome IV criteria were used to ascertain IBS status [23, 24]. Individuals who fulfilled the criteria for IBS were categorized as “individuals with IBS”, whereas those who did not were categorized as “individuals without IBS” (non-IBS). The Bristol Stool Scale (BSS) was used to classify into IBS-subtype: constipation-predominant (IBS-C), diarrhoea-predominant (IBS-D), mixed constipation and diarrhoea (IBS-M), and bowel habits that could not be accurately categorized (IBS-U) [25].

The IBS Severity Scoring System (IBS-SSS) was used to evaluate the severity of IBS-like symptoms [26]. The participants reported their symptoms on a visual analogue scale (0–100), receiving a total sum score between 0 and 500. A higher score indicates more severe symptoms. Based on the IBS-SSS sum score the individuals were categorized into no IBS-like symptoms (< 75), mild IBS-like symptoms (75–175), moderate IBS-like symptoms (175–300) and severe IBS-like symptoms (> 300) [26].

### Faecal samples

One faecal sample was collected from each participant. They received oral and written instruction on how to collect the sample and were instructed to collect and immediately freeze the faecal sample at home. The samples were collected by research staff and stored at − 80 °C until analysis. The samples were analysed for SCFAs at Unger-Vetlesen Institute, Lovisenberg Hospital, Oslo. Upon analysis, the faecal material (0.5 g) was added to distilled water containing 3 mmol/l of 2-ethylbutyric acid (as internal standard) and 0.5 mmol/l of H_2_SO_4_. The faecal material was then homogenized, and 2.5 ml of the homogenate was vacuum distilled. The distillate was analysed through gas chromatography (Agilent 7890 A, USA) using a capillary column and quantified using internal standardization. Flame ionization detection was employed [27, 28]. The results are expressed in mmol/kg wet weight. The major (acetic acid, propionic acid and butyric acid) and minor SCFAs (isobutyric acid, valeric acid, isovaleric acid, caproic acid, and isocaproic acid) were analysed.

### Dietary assessment and anthropometric measurements

A validated 279-item semiquantitative self-administered web-based food frequency questionnaire (WebFFQ) was used to estimate the dietary intake [29, 30]. Briefly, the participants reported the frequency of consumption (ranging from daily to monthly basis) from a list of specific foods or food types and collected information on typical portion sizes with pictures showing the different portion sizes. In addition, it included questions about the intake of dietary supplements. Dietary data was calculated using the food database “KostBeregningsSystemet” (KBS, version 7.4, database AE-18, Department of Nutrition, University of Oslo, Norway). In the data analysis, the dietary supplements intake is included in the nutrient calculation.

### Statistical analyses

The statistical analyses were performed using R v.4.3.0 (R Foundation for Statistical Computing, Vienna, Austria, https://www.r-project.org) and SPSS IBM Statistics (Version 31.0.1.0 (49), IBM Corp, Armonk, NY, USA). The continuous variables are presented as mean and standard deviation. The Shapiro–Wilk test was used to determine whether variables were normally distributed. Unpaired t-test (one-way ANOVA for more than two groups) was used to assess statistical comparisons for normally distributed variables. Non-normally distributed variables were log-transformed. If this improved the distribution, an unpaired t-test (one-way ANOVA for more than two groups) was used on the log-transformed variables. A non-parametric Mann–Whitney U test, or Kruskal–Wallis test for more than two groups, were used for non-normal distributed data not improved by log-transformation. Moreover, categorical variables were expressed as frequency and percentage and compared using the chi-square test, and associations were explored via the Spearman’s rank correlation. Furthermore, we performed a multivariate linear regression analysis to further assess the association between GI symptoms (IBS-SSS and GSRS) and faecal SCFAs. The models were adjusted for potential confounders, including total energy intake (kJ/day), dietary fibre intake (energy-adjusted, g/1000 kcal), sex and BMI (kg/m^2^). In all analyses, a *p*-value of < 0.05 was considered statistically significant.

In the dietary data analysis, we excluded participants (n = 69) who had a daily energy intake lower or higher than set levels (females: < 700 kcal/day or > 3600 kcal/day; males: < 800 kcal/day or > 4200 kcal/day), based on previous HUSK-studies [31]. The multivariate nutrient density method was used for energy adjustments, presenting variables as g/1000 kcal (foods and micronutrients) or energy percent (macronutrients) [32]. We explored the correlation between GI and/or IBS symptoms with eight selected food groups: grain products, vegetables, fruit and berries, meat, fish, egg, dairy products, and butter/oil.

## Results

### Demographic data

The participant characteristics are summarized in Table 1. We included 907 individuals aged 66–69 years, where 50.3% were females. The mean BMI score was 26.4 ± 4.1 kg/m^2^, with 59.2% categorized as overweight or obese. Males had a significantly higher BMI score (*p* < 0.001) and a higher prevalence of diabetes type 2 (*p* = 0.004) (self-reported), when compared to women.

**Table 1:** Characteristics of the participants in the Hordaland Health Study 3—the microbiota study (n = 907). Data are shown for the overall population and stratified by sex

Characteristics	Overall	Females	Males	*p *value
Gender, n (%)	907	456 (50.3)	451 (49.7)	–
Age, years	68.2 ± 0.6	68.2 ± 0.6	68.3 ± 0.6	0.545
Height, cm	171.3 ± 9.2	164.5 ± 6.3	178.3 ± 6.0	< 0.001*
Weight, kg	77.5 ± 14.7	70.0 ± 12.8	85.1 ± 12.6	< 0.001*
BMI, kg/m^2^	26.4 ± 4.1	25.9 ± 4.5	26.8 ± 3.6	< 0.001*
Obesity, n (%)	149 (16.5)	73 (16.1)	76 (17.0)	–
Overweight, n (%)	384 (42.6)	162 (35.7)	222 (49.7)	–
Normal weight, n (%)	357 (39.6)	209 (46.0)	148 (33.1)	–
Underweight, n (%)	11 (1.2)	10 (2.2)	1 (0.2)	–
Alcohol consumers^a^, n (%)	230 (80.7)	106 (80.3)	124 (81.0)	0.874
Smokers^a,b^, n (%)	48 (6.6)	27 (7.5)	21 (5.7)	0.371
Diabetes^c^, n (%)	59 (6.5)	19 (4.2)	40 (8.9)	0.004*

Continuous variables are expressed as mean ± standard deviation (SD). Other variables are given as number (n) and percentage (%). ^a^Questionnaire regarding tobacco use and alcohol consumption employed a binary response format, requiring participants to respond with either 'yes' or 'no.' ^b^Based on *n* = 726, *n* = 182 missing values (*n* = 84 missing male, *n* = 98 missing female. ^c^Defined based on one study measurement of Hba1c, diabetes defined as Hba1c > 48 mmol/L, *n* = 901, 6 missing. Asterisk (*) indicates statistical significance (*p* < 0.05). Abbreviations BMI, body mass index; E%: energy percentage

In Table 2**,** the dietary food intake calculated from the WebFFQ is presented. The overall mean energy intake was 2467 ± 715 kcal/day. Males had a significantly higher total energy intake compared to females (*p* < 0.001). Overall, carbohydrates contributed on average 40.5 energy percent (E%) to the energy intake, fat 36.0 E%, and proteins 18.4 E%. The mean dietary fibre intake was 12.9 ± 4.5 g/1000 kcal. Males had significantly higher intake of carbohydrates (E%) (*p* = 0.007) and dietary fibre (g/1000 kcal) compared to females. Females had a significantly higher fat intake (E%) compared to males (*p* = 0.001).

**Table 2:** Dietary characteristics of participants (age 66–69 years) in the Hordaland Health Study 3 (HUSK3)—Microbiota Study. Data are shown for the overall population and stratified by sex

Characteristics	Overall (*n* = 649)	Females (*n* = 327)	Males (*n*= 322)	*p *value
Total energy intake (kcal/d)	2467 ± 715	2184 ± 616	2754 ± 692	< 0.001^*^
Carbohydrate, E%	40.5 ± 6.2	39.9 ± 6.5	41.1 ± 5.9	< 0.007^*^
Fiber, g/1000 kcal	12.9 ± 4.5	12.3 ± 4.4	13.4 ± 4.4	0.001^*^
Fat, E%	36.0 ± 5.7	36.7 ± 5.9	35.3 ± 5.4	0.001^*^
Protein, E%	18.4 ± 2.7	18.5 ± 2.7	18.3 ± 2.7	0.532
*Intake of major food groups (g/1000 kcal)*				
Grain products	35.7 ± 31.4	31.2 ± 24.5	40.2 ± 36.7	0.004^*^
Vegetables	154.4 ± 92.6	169.5 ± 98.3	139.1 ± 83.9	< 0.001^*^
Fruit and berries	138.9 ± 93.8	134.3 ± 86.9	143.5 ± 100.2	0.290
Meat	48.5 ± 24.2	40.1 ± 18.5	56.9 ± 26.2	< 0.001^*^
Fish	49.4 ± 25.7	42.7 ± 21.1	56.3 ± 28.0	< 0.001^*^
Eggs	10.9 ± 7.3	9.7 ± 7.1	10.2 ± 7.5	0.483
Dairy products	113.7 ± 103.5	102.7 ± 95.9	124.8 ± 109.7	0.022^*^
Butter	13.8 ± 9.9	10.9 ± 8.4	16.7 ± 10.4	< 0.001^*^

Continuous variables are expressed as mean ± standard deviation (SD). Other variables are given as number (n) and percentage (%). Asterisk (*) indicates statistical significance (*p* < 0.05). E%, energy percentage

Regarding selected food groups, males consumed higher amounts of grain products (*p* = 0.004), meat (*p* < 0.001), fish (*p* < 0.001), dairy products (*p* = 0.022) and butter (*p* < 0.001). The intake of vegetables was significant higher in females (*p* < 0.001).

### Gastrointestinal symptoms and prevalence of IBS

The GI symptoms (according to GSRS) and IBS-severity score (based on IBS-SSS) are shown in Table 3**.** In total, 33.9% of the participants reported that they experienced at least one GI symptoms in the past week. In the whole group, 24.2% reported to experience indigestion, 12.0% constipation, 10.3% diarrhoea, 7.6% reflux and 5.7% abdominal pain. Females had a significantly higher mean score of total GI symptoms compared to males and reported significantly higher frequency of constipation and abdominal pain, than males (*p* = 0.034, *p* = 0.028, respectively).

**Table 3:** Self-reported gastrointestinal symptoms (GSRS) and IBS-like symptoms (IBS-SSS) in participants (age 66–69 years) in the Hordaland Health Study 3 (HUSK3) – Microbiota Study. Data are shown for the overall population and stratified by sex

	Overall	Female	Male	*p *value
GI symptoms^a^	*n* = 865	*n *= 434	*n *= 431	
Total mean score	1.7 ± 0.7	1.8 ± 0.7	1.7 ± 0.6	0.021
Abdominal pain	1.5 ± 0.7	1.6 ± 0.7	1.5 ± 0.6	0.031*
Reflux	1.6 ± 0.7	1.6 ± 0.8	1.6 ± 0.7	0.982
Indigestion	2.2 ± 1.0	2.2 ± 1.0	2.1 ± 1.0	0.529
Diarrhea	1.7 ± 1.0	1.7 ± 1.0	1.6 ± 0.8	0.137
Constipation	1.7 ± 0.9	1.8 ± 1.0	1.6 ± 0.8	0.006*
Have at least one GI symptom, n (%)	294 (33.9)	155 (35.0)	139 (32.3)	0.213
Abdominal pain, n (%)	50 (5.7)	33 (7.4)	17 (3.9)	0.023*
Reflux, n (%)	67 (7.6)	40 (9.1)	27 (6.2)	0.109
Indigestion, n (%)	211 (24.2)	106 (24.1)	105 (24.2)	0.956
Diarrhea, n (%)	90 (10.3)	51 (11.6)	39 (9.0)	0.197
Constipation, n (%)	105 (12.0)	65 (14.8)	40 (9.2)	0.012*

IBS-like symptoms^b^	*n* = 881	*n* = 437	*n* = 444	
IBS-SSS Sum score	35.2 ± 54.9	38.4 ± 60.2	31.9 ± 48.7	0.132
No symptoms, n (%)	736 (83.5)	364 (82.0)	372 (85.1)	–
Mild, n (%)	107 (12.1)	54 (12.2)	53 (12.1)	–
Moderate, n (%)	37 (4.2)	25 (5.6)	12 (2.7)	–
Severe, n (%)	1 (0.1)	1 (0.2)	0 (0.0)	–

The data are expressed as mean ± standard deviation or number and percentage. ^a^Data from symptoms

questionnaire GSRS. GI symptom based on GSRS with cut-off ≥ 3. ^b^Data from symptoms questionnaire IBS-SSS. IBS severity based on the IBS-SSS sum score: mild (75–175), moderate (175–300) and severe (> 300). Asterisk (*) indicates statistical significance (*p *< 0.05). IBS, irritable bowel syndrome; IBS-SSS, irritable bowel syndrome-scoring system; GSRS, gastrointestinal symptoms rating scale

Within the study population, 16.4% (*n* = 145) reported IBS-like symptoms (IBS-SSS > 75 points), whereof 12.1% had mild, 4.2% moderate and 0.1% severe IBS-like symptoms. We observed no statistically significant differences between gender for IBS-severity classifications (*p* = 0.132). The overall prevalence of IBS according to Rome IV criteria was 5.0% (*n* = 43), and we found no statistically significant difference between females and males (5.3% vs. 4.7%, respectively, *p* = 0.667). Of the individuals diagnosed with IBS (*n* = 43), 35.0% had IBS-M, 30.0% had IBS-D and 25.0% had IBS-C. The mean IBS-SSS sum score in this sub-population (*n* = 43) was 105.5 ± 102.4, whereof 7 had mild, 14 had moderate and 1 had severe IBS-like symptoms. Individuals with IBS (*n* = 43) had a significantly higher IBS-SSS sum score compared to individuals without IBS (*n* = 816) (*p* < 0.001). In the whole study population, we observed no significant differences between males and females for the IBS-SSS sub scores (*p* = 0.143).

### Concentrations of faecal short-chain fatty acids

We observed that concentrations of total and all the major faecal (acetic, propionic and butyric) SCFAs were significantly higher in males than females (Table 4). For the minor faecal SCFAs, the concentration of valeric acid was significantly higher in males than females (*p* < 0.001). We observed no significant differences in the concentrations of any of the minor faecal SCFAs when comparing gender, or individuals with IBS compared to those without (Table 4).

**Table 4:** Concentration of faecal short-chain fatty acids (SCFAs) (mmol/kg wet weight) in participants in the Hordaland Health Study 3—microbiota study.

	Overall	Female	Male	*p *value	IBS^a^	Non-IBS^a^	*p *value
	*n* = 851	*n* = 424	*n* = 427		*n* = 43	*n* = 814	
Total SCFAs	54.7 ± 25.2	52.2 ± 24.3	57.3 ± 25.9	0.004^*^	58.3 ± 30.4	54.6 ± 24.9	0.627
*Major SCFAs*							
Acetic acid	29.3 ± 14.2	28.2 ± 14.1	30.4 ± 14.3	0.021^*^	31.1 ± 16.4	29.3 ± 14.1	0.654
Propionic acid	9.31 ± 4.88	8.80 ± 4.67	9.82 ± 5.05	0.001^*^	9.76 ± 4.83	9.29 ± 4.89	0.570
Butyric acid	10.2 ± 6.64	9.60 ± 6.20	10.9 ± 7.01	0.002^*^	11.70 ± 9.56	10.2 ± 6.46	0.559
*Minor SCFAs*							
Valeric acid	1.59 ± 0.84	1.48 ± 0.75	1.71 ± 0.91	< 0.001^*^	1.61 ± 0.72	1.60 ± 0.85	0.766
Isovaleric acid	2.15 ± 1.29	2.10 ± 1.16	2.30 ± 1.40	0.080	1.94 ± 0.99	2.17 ± 1.30	0.522
Isobutyric acid	1.48 ± 0.81	1.42 ± 0.73	1.54 ± 0.88	0.061	1.34 ± 0.62	1.49 ± 0.81	0.466
Caproic acid	0.64 ± 0.82	0.60 ± 0.75	0.70 ± 0.90	0.066	0.86 ± 1.01	0.63 ± 0.81	0.204
Isocaproic	0.01 ± 0.03	0.01 ± 0.03	0.01 ± 0.04	0.101	0.01 ± 0.02	0.01 ± 0.03	0.320

The results are given for the overall population, stratified by sex and IBS-status according to Rome IV criteria

Data expressed as mean ± standard deviation or percentage. The concentration of faecal SCFAs is given in mmol/kg wet weight. ^a^The Rome IV criteria were used to ascertain IBS status. Individuals who fulfilled the criteria for IBS were categorized as “individuals with IBS”, whereas those who did not were categorized as “individuals without IBS” (non-IBS). Asterisk (*) indicates statistical significance (*p* < 0.05). IBS, irritable bowel syndrome; SCFAs, short-chain fatty acids

### Correlations between GI symptoms and associated factors

The correlations between GI symptoms (according to IBS-SSS, GSRS and BSS) and concentration of faecal SCFAs are presented in Table 5. Furthermore, the results from the multivariate linear regression analyses between GI symptom scores (IBS-SSS and GSRS) and faecal SCFAs are presented in Table 6. Total faecal SCFAs were negatively correlated with constipation (*r* =  − 0.188, *p* < 0.001). This association remained significant in the regression analysis, both in the crude model (Unstandardized beta coefficient =  − 0.006, 95% CI [− 0.009, − 0.004], *p* < 0.001) and after adjustment for dietary fibre intake, total energy intake, sex and BMI (Unstandardized beta coefficient =  − 0.006, 95% CI [− 0.009, − 0.004], *p* < 0.001) (Table 6). A positive correlation was observed between the concentration of total faecal SCFAs and looser stool consistency (*r* = 0.185, *p* < 0.001) and diarrhoea (*r* = 0.072, *p* = 0.039) (Table 5**).** The positive association between total faecal SCFA and diarrhoea did not persist in the regression analysis, neither in the crude or adjusted models **(**Table 6).

**Table 5:** Spearman’s correlation coefficient between gastrointestinal symptoms and faecal short chain fatty acids (SCFAs) (mmol/kg wet weight, absolute numbers) in participants (age 66–69 years) in the Hordaland Health Study 3 (HUSK3)—microbiota study

R-value							
	IBS-SSS	Abdominal pain (GSRS)	Reflux (GSRS)	Indigestion (GSRS)	Diarrhea (GSRS)	Constipation (GSRS)	Stool consistency (BSS)
Total SCFAs	r: 0.051	r: − 0.014	r: − 0.020	r: − 0.021	r:0.072^*^	r: − 0.188^***^	r: 0.185^***^
*Major SCFAs*							
Acetic acid	r:0.096^**^	r: − 0.021	r: − 0.021	r: − 0.022	r: 0.090^**^	r: − 0.188^***^	r: 0.197^***^
Propionic acid	r: 0.009	r: 0.009	r: − 0.013	r: − 0.039	r: 0.089^*^	r: − 0.176^***^	r: 0.179^***^
Butyric acid	r: − 0.025	r: − 0.019	r: − 0.010	r: − 0.008	r: 0.055	r: − 0.830^***^	r: 0.174^***^
*Minor SCFAs*							
Valeric acid	r: − 0.019	r: − 0.031	r: − 0.020	r: 0.000	r: − 0.040	r: − 0.960^**^	r: 0.058
Isovaleric acid	r: − 0.023	r: − 0.028	r: 0.001	r: − 0.011	*r:* − 0.171^***^	r: 0.059	r: − 0.149^***^
Isobutyric acid	r: − 0.028	r: − 0.033	r: − 0.004	r: − 0.015	r: − 0.143^***^	r: 0.012	r: − 0.101^**^
Caproic acid	r: − 0.044	r: − 0.051	r: − 0.020	r: 0.025	r: − 0.060	r: − 0.091^**^	r: 0.044
Isocaproic acid	r: 0.032	r: 0.056	r: 0.048	r: 0.036	r: 0.051	r: 0.038	r: 0.069

^*^p < 0.05, **p < 0.01, ***p < 0.001 level. SCFAs, short-chain fatty acids; GSRS, gastrointestinal symptoms rating scale; IBS, irritable bowel syndrome; BSS, Bristol stool scale; IBS, irritable bowel syndrome; IBS-SSS, irritable bowel syndrome-severity scoring system

**Table 6:** Association between faecal short chain fatty acid levels (mmol/L wet weight) and gastrointestinal symptoms measured by symptom severity (IBS-SSS) and gastrointestinal rating scale (GSRS) in participants (age 66–69 years) in the Hordaland Health Study 3 (HUSK3)—Microbiota study

		IBS-SSS		Abdominal pain (GSRS)		Reflux (GSRS)		Indigestion (GSRS)		Diarrhoea (GSRS)		Constipation (GSRS)	
Exposure	Model	Estimate (95% CI)	*p *value		*p *value		*p *value		*p *value		*p *value		*p *value
Total SCFA	M1	0.061 (− 0.095, 0.216)	0.444	− 0.001 (− 0.003, 0.001)	0.317	− 0.001 (− 0.004, 0.001)	0.238	0.000 (− 0.003, 0.003	0.778	0.002 (0.001, 0.005)	0.195	*** − ****0.006 (****− ****0.009, − 0.004)*	* < 0.001*
	M2	0.071 (− 0.087, 0.229)	0.380	− 0.001 (− 0.003, 0.001)	0.219	− 0.002 (− 0.004, 0.00)	0.062	− 0.001 (− 0.004, 0.002)	0.569	0.002 (− 0.001, 0.005)	0.167	*** − ****0.006 (****− ****0.009, − 0.004)*	* < 0.001*
*Major SCFA*													
Acetic acid	M1	0.269 (− 0.009, 0.547)	0.058	− 0.002 (− 0.005, 0.002)	0.335	− 0.003 (− 0.007, 0.001)	0.204	0.000 (− 0.005, 0.006)	0.898	0.005 (0.000, 0.010)	0.050	*** − ****0.012 (****− ****0.016, − 0.007)*	* < 0.001*
	M2	**0.286 (0.004, 0.568)**	**0.047**	− 0.002 (− 0.006, 0.001)	0.240	− 0.004 (− 0.008, 0.000)	0.060	0.000 (− 0.006, 0.005)	0.912	**0.005 (0.000, 0.010)**	**0.043**	*** − ****0.012 (****− ****0.017, − 0.007)*	* < 0.001*
Propionic acid	M1	− 0.096 (− 0.894, 0.702)	0.813	− 0.005 (− 0.016, 0.005)	0.310	− 0.008 (− 0.020, 0.003)	0.167	− 0.013 (− 0.028, 0.003)	0.105	**0.015 (0.001, 0.029)**	**0.037**	* − 0.031 (− 0.045, − 0.017)*	* < 0.001*
	M2	− 0.61 (− 0.875, 0.753)	0.883	− 0.007 (− 0.017, 0.004)	0.201	* − 0.013 (− 0.025, − 0.01)*	*0.032*	− 0.015 (− 0.31, 0.000)	0.050	**0.016 (0.002, 0.030)**	**0.029**	* − 0.031 (− 0.046, − 0.017)*	* < 0.001*
Butyric acid	M1	− 0.094(− 0.690, 0.502)	0.756	− 0.02 (− 0.010, 0.005)	0.540	− 0.002 (− 0.011, 0.006)	0.603	0.001 (− 0.011, 0.012)	0.905	0.003 (− 0.008, 0.013)	0.625	* − 0.021 (− 0.031, − 0.010)*	* < 0.001*
	M2	− 0.060 (− 0.663, 0.544)	0.846	− 0.003 (− 0.011, 0.005)	0.460	− 0.004 (− 0.013, 0.004)	0.319	− 0.001 (− 0.012, 0.011)	0.923	0.003 (− 0.007, 0.014)	0.551	* − 0.020 (− 0.031, − 0.010)*	* < 0.001*
*Minor SCFAs*													
Valeric acid	M1	− 3.071 (− 7.716, 1.573)	0.195	− 0.045 (− 0.106, 0.016)	0.148	− 0.031 ( − 0.101, 0.038)	0.371	− 0.038 (− 0.127, 0.050)	0.396	− 0.067 (− 0.150, 0.016)	0.113	* − 0.110 (− 0.193, − 0.026)*	*0.010*
	M2	− 2.950 (− 7.710, 1.810)	0.224	− 0.053 (− 0.115, 0.009)	0.093	− 0.057 (− 0.127, 0.012)	0.107	− 0.054 (− 0.145, 0.036)	0.240	− 0.066 (− 0.150, 0.019)	0.128	* − 0.107 (− 0.192, − 0.022)*	*0.013*
Isovaleric	M1	− 1.523 (− 4.493, 1.448)	0.315	− 0.009 (− 0.047, 0.029)	0.644	− 0.005 (− 0.048, 0.039)	0.832	− 0.013 (− 0.070, 0.043)	0.640	* − 0.083 (− 0.136, − 0.031)*	*0.002*	0.031 ( − 0.022, 0.084)	0.251
	M2	− 1.431 (− 4.438, 1.576)	0.350	− 0.012 (− 0.051, 0.026)	0.530	− 0.014 (− 0.057, 0.029)	0.526	− 0.020 (− 0.077, 0.036)	0.481	* − 0.085 (− 0.138, − 0.033)*	*0.001*	0.034 ( − 0.019, 0.087)	0.205
Isobutyric	M1	− 2.980 (− 7.772, 1.762)	0.218	− 0.018 (− 0.080, 0.043)	0.556	− 0.010 (− 0.080, 0.059)	0.771	-0.029 (− 0.119, 0.060)	0.519	* − 0.111 (− 0.195, − 0.027)*	*0.010*	0.007 (-0.077, 0.092)	0.862
	M2	− 2.854 (− 7.669, 1.961)	0.245	− 0.025 (− 0.087, 0.037)	0.421	− 0.029 (− 0.098, 0.041)	0.415	− 0.043 (− 0.134, 0.048)	0.355	* − 0.115 (− 0.199, − 0.031)*	*0.007*	0.012 (-0.074, 0.097)	0.791
Caproic acid	M1	− 2.309 (− 7.246, 2.628)	0.359	− 0.015 (− 0.080, 0.049)	0.645	− 0.020 (− 0.093, 0.054)	0.596	0.005 (− 0.089, 0.099)	0.918	− 0.043 (− 0.131, 0.046)	0.342	* − 0.125 (− 0.214, − 0.037)*	*0.006*
	M2	− 2.294 (− 7.272, 2.683)	0.366	− 0.013 (− 0.078, 0.051)	0.686	− 0.022 (− 0.95, 0.51)	0.551	0.004 (− 0.091. 0.099)	0.939	− 0.037 (− 0.125, 0.052)	0.417	* − 0.113 (− 0.202, − 0.024)*	*0.012*

All estimates represent unstandardized B-coefficient from a multivariate linear regression analysis. Analysis is based on *n* = 608 participants with complete data for all variables. 95% CI, 95% confidence interval. *Statistically significant association, *p* < 0.05

M1 Crude model

M2 Model adjusted for dietary fibre intake (energy-adjusted, g/1000 kcal), estimated total energy intake (kJ/day), BMI (kg/m^2^) and sex

SCFAs, short-chain fatty acids; GSRS, gastrointestinal symptoms rating scale; IBS, irritable bowel syndrome; BSS, bristol stool scale; IBS, irritable bowel syndrome; IBS-SSS, irritable bowel syndrome-severity scoring system

For the major faecal SCFAs, a positive correlation was observed between IBS-severity (IBS-SSS) and acetic acid (*r* = 0.096, *p* = 0.005) (Table 5). In the linear regression model, acetic acid was not significantly associated with IBS-severity in crude model but became significant after adjustment for confounders (Table 6**).** Diarrhoea showed a positive correlation with acetic acid (*r* = 0.090, *p* = 0.009) and propionic acid (*r* = 0.089, *p* = 0.010) (Table 5). In the linear regression analyses, the association remained significant in both crude and adjusted model for propionic acid, and in the adjusted model only for acetic acid (Table 6). We observed no significant association between diarrhoea and butyric acid (Table 6). A negative association was also observed for reflux and propionic acid in the adjusted regression model (Table 6). Constipation showed a negative correlation with acetic acid (*r* =  − 0.188, *p* < 0.001), propionic acid (*r* =  − 0.176, *p* < 0.001) and butyric acid (*r* =  − 0.830, *p* < 0.001), and these association were also observed in the regression model (Table 6). Looser stool consistency showed a positive correlation with acetic acid (*r* = 0.197 *p* < 0.001), butyric acid (*r* = 0.174, *p* < 0.001) and propionic acid (*r* = 0.179, *p* < 0.001) (Table 5).

For the minor faecal SCFAs, constipation was negatively correlated with valeric acid (*r* =  − 0.960, *p* = 0.006) and caproic acid (*r* =  − 0.091, *p* = 0.009) (Table 5). Diarrhoea was negatively correlated with isovaleric acid (*r* =  − 0.171, *p* < 0.001) and isobutyric acid (*r* =  − 0.143, *p* < 0.001) (Table 5). All these association remained significant in the regression models (Table 6). Further, stool consistency was negatively correlated with isovaleric acid (*r* =  − 0.149, *p* < 0.001) and isobutyric acid (*r* =  − 0.101, *p* < 0.001). All GI symptoms (abdominal pain (*r* = 0.012, *p* < 0.01), reflux (*r* = 0.17, *p* < 0.01), indigestion (*r* = 0.09, *p* = 0.01, constipation (*r* = 0.11, *p* < 0.01) and diarrhoea (*r* = 0.07, *p* = 0.04) showed a weak positive correlation with increasing BMI.

The correlation between dietary factors and GI symptoms, stool consistency (BSS), and IBS-severity (IBS-SSS) can be found in Supplementary Table 4. After energy adjustment, no correlations were found between GI/IBS symptoms and total energy intake. For the macronutrients, we found a negative correlation between protein intake and reflux symptoms (*r* =  − 0.102, *p* = 0.010) (Supplementary Table 4).

## Discussion

To the best of our knowledge, this is the first study exploring the prevalence of GI symptoms and IBS, and their associations with faecal SCFAs, dietary intake and BMI in Norwegian community-dwelling older adults. Our results demonstrate that GI symptoms are common in older adults, with 33.9% experienced at least one GI symptom in the past week, 16.4% reported IBS-like symptoms and 5.0% fulfilled the criteria for IBS according to Rome IV. The most frequent GI symptoms were indigestion, followed by constipation and diarrhoea. We found that females reported a higher total mean score of GI symptoms compared to males. Of older individuals with IBS, the majority had moderate IBS symptoms, with IBS-M as the most common sub-type. We observed that males had significantly higher concentrations of total faecal SCFAs, and all the major SCFAs (acetic, propionic, and butyric acid), and valeric acid compared to females, but we found no differences when comparing those with IBS and without IBS. The concentration of total faecal SCFAs were negatively associated with constipation, independently of dietary fibre intake, total energy intake, sex, and BMI. Further, we observed a negative correlation between reflux and protein intake, whereas indigestion was positively correlated with the average intake of cereals, and loose stool consistency was negatively correlated with intake of dairy products. Additionally, all GI symptoms (according to GSRS) were positively correlated with an increasing BMI.

Other recent epidemiological studies have reported a high prevalence of GI symptoms among community-dwelling older adults [10, 11]. A recent Swedish study in community-dwelling older adults (*n* = 241) reported that 65% of the individuals had self-reported GI symptoms in the past week [11], whereas a Chinese community-based study (*n* = 688) in older adults found that 83.3% of the participants reported at least one GI symptoms in the last six month [10]. In contrast, we found that 33.9% experienced at least one GI symptom in the past week, which is substantially lower compared to the findings presented above [10, 11]. The differences in the prevalence of GI symptoms might be explained by methodological differences, such as differences in chosen cut-off levels. There are currently no standardized cut-off levels for GSRS, and in the study by Zhao et al. [10] they ranked GSRS as a continuous variable, whereas a specific cut-off levels was used in our population and by Fart et al. [11]. In addition, dietary habits and geographical differences may also contribute to different results. Furthermore, the reported prevalences may also have been influence by selection bias. For instance, the Swedish study may have attracted individuals with GI symptoms, since one of their objectives was to explore GI health [11]. Additionally, differences in underlying health status may also be a factor contributing the observed difference in GI symptoms. In the Chinese study [10], they reported that 20.9% of their participants had diabetes type 2, in comparison to 6.5% in our population. A recent study found that older adults with type 2 diabetes were more likely to have GI symptoms, especially constipation, compared to those with normoglycemia [33]. Overall, despite differences in overall prevalence, our results are consistent with those reported by Fart et al. [11], when comparing total GSRS-score and subscale scores, including abdominal pain, reflux, indigestion, diarrhoea and constipation.

We observed that females reported a higher prevalence of constipation and abdominal pain than males, which is in line with previous epidemiological studies [12, 33]. The difference between genders might be due to the significantly lower total intake of dietary fibre observed in females compared to males in our study population, as a lower dietary fibre intake is associated to a slower transit time and an increased risk of constipation [34]. Although the association between constipation and low dietary fibre intake is well-established, it is also demonstrated that constipation may be caused by a combination of several factors, such as low fluid intake, lack of exercise and use of analgesics, in particular opioids [35]. In the present study, similar to Fart et al. [11], we also observed that females experienced more abdominal pain than males. Some studies have found that pain sensitivity varies between males and females, with females reporting more chronic pain conditions, and that females are twice as likely to be prescribed opioids compared to males [36, 37]. In our cohort, we observed a weak negative association between constipation and total SCFA, as well as the major SCFAs (acetic, propionic, and butyric acid) (Table 5), which was present in both men and women in sex-stratified correlation analysis (Supplementary Table 2). Importantly, in the regression analyses, these associations remained unchanged after adjusting for dietary fibre, total energy intake, BMI and sex. This suggests that the link between constipation and reduced levels of total and major SCFAs is not solely due to dietary habits or physiological sex differences. It should also be noted that sex differences in gut microbiota composition [38] may influence the observed differences in SCFAs levels and GI symptoms. Interestingly, our population consist of older adults, and studies have shown that the gut microbiota composition after menopause becomes more similar the composition observed in men [39]. However, gut microbiota composition was not assessed in this study, so this should be considered when interpreting the findings and should be further assessed in future studies.

According to the Rome IV criteria, 5.0% had IBS in our study population of older community-dwelling adults. This is slightly higher compared to the general population, where the IBS prevalence has reported to be 3.8%, using the same criteria [15]. This underlies that older adults also suffer from IBS, despite considered a GI disorder primally affecting young females [40]. A cross-sectional study in Oppland County in Norway estimated the prevalence of IBS to be 8% among individuals aged 30–75 years when using the Rome II criteria [41], whereas more recent cross-sectional study from the Trøndelag Health Study 4 (HUNT4) estimated the prevalence of IBS to be 9.5% in individuals > 20 years, also using the same criteria [16]. In comparison, a community survey of 230 adults aged 65–94 year, 22% had IBS (Rome III) [42], which are a much higher prevalence compared to our results. This might be due to the different Rome criteria used, as the Rome IV criteria are more restrictive compared to previous Rome criteria by removing “abdominal discomfort” from the criteria [24]. Therefore, it is evident that the criteria used to define IBS influence the prevalence estimates of IBS.

There are limited studies assessing the concentration of faecal SCFAs in humans, especially in older adults. However, some recent observational studies have reported an age-associated reduction in levels of faecal SCFAs [43–45]. A cross-sectional study by Salazar et al.

[45] observed that those > 80 years of age had lower concentration of the major SCFAs, with less than half compared to the younger adult group (< 50 years old). When stratified by sex in our study population, males had significantly higher levels of total faecal SCFAs, and all the major SCFAs (acetic, butyric and propionic acids) compared to females. Interestingly, we also observed that males experienced a significantly lower total mean score of GI symptoms, including lower frequency of abdominal pain and constipation compared to females. These sex differences are in line with what other report [11, 46], and might be due to differences in hormones, altered gut motility or visceral sensitivity [47, 48]. Another plausible explanation for the observed difference, may be due to differences in symptom reporting, and it is well known that women more frequently report somatic symptoms compared to men [49]. When it comes to the sex differences observed in levels of the faecal SCFAs, this might be explained by the observed significantly higher intake of dietary fibre in males compared to females in our study, as a diet low in fibre has been associated with a decline in butyric acid [50]. However, these gender differences should be further explored in future studies, as there might be some underlying biological or hormonal differences that may explain some of our findings.

A previous study investigated the association between GI symptoms and dietary factors (consumption of fat, fruit, and fibre) in participants with obesity and observed that fat intake was positively associated with diarrhoea [51]. Other studies have also shown that individuals with GI dysfunction have an increased sensitivity to fat, and lipids may be responsible for the postprandial exacerbation of bloating, nausea, and fullness [52]. We did not observe any association between fat intake and GI symptoms; however, we observed that a higher level of total faecal SCFAs were associated with looser stool consistency, and lower levels of total faecal SCFAs were associated with constipation. We also found that diarrhoea was positively correlated with both acetic acid and propionic acid. Further, we found a weak positive correlation between all GI symptoms (measured by GSRS) and BMI, in line with some previous studies [53], but not others [54]. Murray et al. [53] observed that being overweight significantly increased the frequency of reflux symptoms and indicated that weight reduction might decrease the occurrence of reflux symptoms [53]. One the other side, a cross-sectional study among middle-aged and older adults found no association between reflux symptoms and BMI [54]. The authors concluded that reflux symptoms occur independently of BMI, and that weight reduction may not be justifiable as an anti-reflux therapy [54].

The current study has several strengths and limitations. A considerable strength is the large study population of older adults, and to the best of our knowledge, this is also the first study exploring the prevalence of GI symptoms and IBS using the Rome IV criteria in Norwegian community-dwelling older adults. Several limitations should also be considered when interpreting the findings. Firstly, due to the cross-sectional study design we are not able to determine causality or development over time. Potential causal pathways and clarifications should be assessed in future longitudinal studies. Secondly, data on medication and underlying disease was not collected in this study. Both these factors could affect GI symptoms, either as side effects or as a secondary clinical presentation of a disease, so future studies should include these data for a more thorough analysis. Thirdly, we used a self-administered WebFFQ to measure dietary intake, which may be influenced by recall bias. The accuracy of the data could have been improved by also including other methods for collecting dietary data, which should be considered in future studies. Furthermore, gut microbiota was not analysed, which is a limitation, and these data could have given us additional insights in the observed association and potential underlying mechanistic pathways. Finally, our results might not be generalizable to the entire population of older adults, as the study population consisted of a sub-sample of relatively healthy older adults living in the Bergen region, Norway. Overall, future research should address these methodological considerations, which could provide stronger evidence and clarify the observed associations.

In conclusion, our results indicates that GI symptoms are common, and IBS are at least as prevalent in Norwegian community-dwelling older adults as observed in the general adult population. Females reported more GI symptoms, including constipation and abdominal pain, but did not have a higher prevalence of IBS or more IBS-like symptoms compared to males. In future studies, potential mechanisms driving these sex difference in GI symptoms should be further assessed, including potential biological or hormonal mechanisms. Further, we found that various GI symptoms are associated with faecal SCFAs, dietary intake and BMI. A strategy to reduce the risk of GI symptoms could therefore be to improve dietary habits and to prevent overweight and obesity in the older population. However, these associations should be further explored in future longitudinal and mechanistical studies. This is the first study, to the best of our knowledge, exploring the prevalence of GI symptoms and IBS, and their association with faecal SCFAs, dietary intake and BMI in Norwegian community-dwelling older adults. This fills a knowledge-gap, and our research gives a foundation for future preventive and therapeutic strategies and may guide evidence-based public health policies to promote gut health in the older population. It is crucial to develop strategies to alleviate and prevent GI symptoms in older adults, and addressing gut health in older adults will promote healthy ageing and, ultimately, improve the quality of life for the individual.

## Supplementary Information

Below is the link to the electronic supplementary material.

Supplementary file1 (DOCX 41 KB)

## Data Availability

Data described in the manuscript will be made available pending application and approval.
