# Supplementary material for: Gastrointestinal symptoms, faecal short-chain fatty acids and dietary intake in older adults: the Hordaland Health Study 3
Source: Eur J Nutr. 2026 Aug 6;65(6):230. doi: 10.1007/s00394-026-04077-6 (PMC13447390; doi:10.1007/s00394-026-04077-6)
Supplement: Supplementary file 1 [file 394_2026_4077_MOESM1_ESM.docx]

**Supplementary Material: European Journal of Nutrition**

**Gastrointestinal symptoms, fecal short-chain fatty acids and dietary intake in older adults: The Hordaland Health Study 3**

**Authors:** Tilde Martinsen, Caroline Jensen, Jan Gunnar Hatlebakk, Jørgen Valeur, Ottar Nygård, Gülen Arslan Lied

**Corresponding author:**

**Corresponding author:** Caroline Jensen, Department of Medicine, Haukeland University Hospital, 5021 Bergen, Norway, E-mail: [caroline.j@uib.no](mailto:caroline.j@uib.no)

**List of materials included in the Supplementary Information:**

**Supplementary Tables**

**Supplementary Table 1** Short-chain fatty acids (SCFAs) (% of total SCFA) in participants in the Hordaland Health Study 3 – Microbiota Study. The results are given for the overall population, stratified by sex and IBS-status according to Rome IV criteria.

**Supplementary Table 2** Sex-specific Spearman’s correlation coefficient between gastrointestinal symptoms and faecal short chain fatty acids (SCFAs) (mmol/kg wet weight, absolute numbers) in participants (age 66-69 years) in the Hordaland Health Study 3 (HUSK3) – Microbiota study.

**Supplementary Table 3** Spearman’s correlation coefficient between dietary factors and gastrointestinal symptoms in participants (age 66-69 years) in the Hordaland Health Study 3 (HUSK3) – Microbiota study.

**Supplementary Table 4.** Spearman’s correlation coefficient between dietary factors and gastrointestinal symptoms in participants (age 66-69 years) in the Hordaland Health Study 3 (HUSK3) – Microbiota study.

**Supplementary** **Table 1.** Short-chain fatty acids (SCFAs) (% of total SCFA) in participants in the Hordaland Health Study 3 – Microbiota Study. The results are given for the overall population, stratified by sex and IBS-status according to Rome IV criteria.

|  | | **Overall** | **Female** | **Male** | **P-value** | IBS | Non-IBS | P-value | |  |
| --- | --- | --- | --- | --- | --- | --- | --- | --- | --- | --- |
|  | | *n*=851 | *n=*424 | *n*=426 |  | *n*=43 | *n*=814 |  | |  |
| **Total SCFAs** | | 54.7 ± 25.2 | 52.2 ± 24.3 | 57.3 ± 25.9 | 0.004* | 58.3 ± 30.4 | 54.6 ± 24.9 | 0.627 | |  |
| ***Major SCFAs*** | |  |  |  |  |  |  |  | |  |
|  | Acetic acid | 53.4 ± 7.7 | 53.9 ± 7.8 | 52.9 ± 7.7 | 0.075 | 52.9 ± 7.2 | 53.4 ± 7.8 | 0.634 |  |  |
|  | Propionic acid | 17.2 ± 4.2 | 17.0 ± 4.1 | 17.4 ± 4.4 | 0.441 | 17.2 ± 4.6 | 17.2 ± 4.3 | 0.999 |  |  |
|  | Butyric acid | 17.7 ± 5.0 | 17.4 ± 5.2 | 18.1 ± 4.9 | 0.023* | 17.7 ± 5.0 | 16.9 ± 4.8 | 0.417 |  |  |
| ***Minor SCFAs*** | |  |  |  |  |  |  |  | |  |
|  | Valeric acid | 3.1 ± 1.1 | 3.0 ± 1.0 | 3.1 ± 1.5 | 0.065 | 3.0 ± 0.8 | 3.1 ± 1.1 | 0.699 |  |  |
|  | Isovaleric acid | 4.5 ± 2.4 | 4.6 ± 2.4 | 4.4 ± 2.4 | 0.248 | 4.2 ± 2.6 | 4.5 ± 2.4 | 0.391 |  |  |
|  | Isobutyric acid | 3.0 ± 1.4 | 3.1 ± 1.4 | 3.0 ± 1.4 | 0.179 | 2.8 ± 1.5 | 3.0 ± 1.4 | 0.300 |  |  |
|  | Caproic acid | 1.2 ± 1.2 | 1.1 ± 1.1 | 1.2 ± 1.3 | 0.502 | 1.3 ± 1.2 | 1.1 ± 1.2 | 0.236 |  |  |
|  | Isocaproic | 0.0 ± 0.1 | 0.0 ± 0.1 | 0.0 ± 0.1 | 0.065 | 0.0 ± 0.0 | 0.0 ± 0.1 | 0.322 |  |  |

Data expressed as mean ± standard deviation or percentage. The concentration of faecal SCFAs is given in mmol/kg wet weight. ^a^The Rome IV criteria were used to ascertain IBS status. Individuals who fulfilled the criteria for IBS were categorized as “individuals with IBS”, whereas those who did not were categorized as “individuals without IBS” (non-IBS). Asterisk (*) indicates statistical significance (p<0.05). Abbreviations; IBS, irritable bowel syndrome; SCFAs, short-chain fatty acids.

**Supplementary Table 2.** Sex-specific spearman’s correlation coefficient between gastrointestinal symptoms and faecal short chain fatty acids (SCFAs) (mmol/kg wet weight, absolute numbers) in participants (age 66-69 years) in the Hordaland Health Study 3 (HUSK3) – Microbiota study.

|  | |  |  |  |  |  |  | **R-value** |  |  |  |  |  |  |  |
| --- | --- | --- | --- | --- | --- | --- | --- | --- | --- | --- | --- | --- | --- | --- | --- |
|  | | **IBS-SSS** | | **Abdominal pain (GSRS)** | | **Reflux (GSRS)** | | **Indigestion (GSRS)** | | **Diarrhea (GSRS)** | | **Constipation (GSRS)** | | **Stool consistency (BSS)** | |
|  | | **F** | **M** | **F** | **M** | **F** | **M** | **F** | **M** | **F** | **M** | **F** | **M** | **F** | **M** |
| **Total SCFAs** | | 0.034 | 0.078 | −0.029 | 0.014 | −0.004 | -0.040 | −0.002 | −0.021 | 0.089 | 0.067 | −**0.207***** | −***0.149***** | **0.239***** | **0.120*** |
| ***Major SCFAs*** | |  |  |  |  |  |  |  |  |  |  |  |  |  |  |
|  | AA | 0.070 | **0.129**** | −0.040 | 0.014 | −0.009 | −0.038 | −0.004 | −0.022 | **0.096** | **0.096*** | −**0.211***** | −**0.144**** | **0.252***** | **0.134*** |
|  | PA | −0.008 | 0.035 | 0.027 | 0.004 | −0.008 | −0.021 | −0.001 | −0.039 | **0.134**** | 0.059 | −**0.189***** | −**0.140**** | **0.265***** | 0.084 |
|  | BA | −0.041 | −0.004 | −0.028 | 0.000 | 0.013 | -0.041 | −0.004 | −0.008 | 0.082 | 0.038 | −**0.214***** | −**0.132**** | **0.229***** | **0.116*** |
| ***Minor SCFAs*** | |  |  |  |  |  |  |  |  |  |  |  |  |  |  |
|  | VA | −0.029 | 0.004 | −0.025 | −0.020 | 0.001 | −0.051 | 0.056 | 0.000 | 0.011 | -0.078 | -0.073 | −**0.098*** | **0.125*** | **-0.014** |
|  | IVA | −0.012 | −0.024 | −0.007 | −0.043 | 0.029 | −0.032 | 0.053 | −0.011 | −0.114* | −**0.230***** | **0.165***** | −0.034 | −**0.186***** | −**0.124*** |
|  | IBA | −0.19 | −0.025 | −0.011 | −0.046 | 0.031 | −0.042 | 0.053 | −0.015 | −0.072 | −**0.213***** | **0.110*** | −0.073 | −**0.125*** | -0.086 |
|  | CA | −0.042 | −0.042 | −0.071 | −0.020 | 0.008 | −0.050 | 0.055 | 0.025 | −0.065 | −0.052 | −0.078 | −0.096 | 0.046 | 0.039 |
|  | ISA | 0.051 | 0.024 | 0.018 | 0.095 | 0.015 | 0.071 | 0.008 | 0.036 | 0.014 | 0.086 | 0.016 | 0.0670 | 0.023 | **0.105*** |

*Correlation is significant at the 0.05 level ** Correlations is significant at the 0.01 level *** Correlation is significant at the 0.001 level Asterisk (*) indicates statistical significance (p<0.05). SCFAs, short-chain fatty acids; GSRS, gastrointestinal symptoms rating scale; IBS, irritable bowel syndrome; BSS, Bristol stool scale; IBS, irritable bowel syndrome; IBS-SSS=irritable bowel syndrome – Severity Scoring System.

**Supplementary Table 3.** Spearman’s correlation coefficient between gastrointestinal symptoms and faecal short chain fatty acids (SCFAs) (% of total) in participants (age 66-69 years) in the Hordaland Health Study 3 (HUSK3) – Microbiota study.

|  | |  |  |  | **R-value** |  |  |  |
| --- | --- | --- | --- | --- | --- | --- | --- | --- |
|  | | **IBS-SSS** | **Abdominal pain (GSRS)** | **Reflux (GSRS)** | **Indigestion (GSRS)** | **Diarrhea (GSRS)** | **Constipation (GSRS)** | **Stool consistency (BSS)** |
|  | |  |  |  |  |  |  |  |
| **Total SCFAs** | | r: 0.059 | r: -0.018 | r: 0.009 | r: -0.002 | r: 0.052 | **r:** −**0.018*** | **r: 0.285*** |
| ***Major SCFAs*** | |  |  |  |  |  |  |  |
|  | Acetic acid | **r: 0.093*** | r: −0.019 | r: −0.007 | r: 0.005 | **r: 0.084*** | **r: −0.180*** | **r: 0.306*** |
|  | Propionic acid | r: 0.031 | r: 0.003 | r: −0.008 | r: −0.032 | **r: 0.076*** | **r: −0.169*** | **r: 0.275*** |
|  | Butyric acid | r: 0.020 | r: −0.015 | r: 0.002 | r: 0.056 | r: 0.014 | **r: −0.154*** | **r: 0.221*** |
| ***Minor SCFAs*** | |  |  |  |  |  |  |  |
|  | Valeric acid | r: −0.023 | r: −0.045 | r: −0.033 | r: −0.016 | r: −0.063 | **r: −0.121*** | **r: 0.124*** |
|  | Isovaleric acid | r: -0.026 | r: −0.014 | r: −0.023 | r: −0.007 | **r: −0.128*** | r: −0.089 | r: −0.108 |
|  | Isobutyric acid | r: −0.033 | r: −0.019 | r: −0.031 | r: −0.013 | **r: −0.108*** | r: −0.028 | r: −0.049 |
|  | Caproic acid | r: −0.038 | r: −0.041 | r: −0.030 | r: 0.020 | r: −0.055 | r: −0.120 | r: 0.167 |
|  | Isocaproic acid | **r: 0.079*** | r: 0.052 | r: 0.042 | r: 0.067 | **r: 0.125*** | r: 0.054 | r: 0.072 |

Asterisk (*) indicates statistical significance (p<0.05). SCFAs, short-chain fatty acids; GSRS, gastrointestinal symptoms rating scale; IBS, irritable bowel syndrome; BSS, Bristol stool scale; IBS, irritable bowel syndrome; IBS-SSS=irritable bowel syndrome – Severity Scoring System

**Supplementary Table 4.** Spearman’s correlation coefficient between dietary factors and gastrointestinal symptoms in participants (age 66-69 years) in the Hordaland Health Study 3 (HUSK3) – Microbiota study.

| **R-value** | | | | | | | | |
| --- | --- | --- | --- | --- | --- | --- | --- | --- |
|  | | **IBS-Severity**  **(IBS-SSS)** | **Abdominal pain (GSRS)** | **Reflux (GSRS)** | **Indigestion (GSRS)** | **Diarrhea (GSRS)** | **Constipation (GSRS)** | **Stool consistency (BSS)** |
| ***Total energy (kcal/day)*** | | r: −0.026 | *r: 0.020* | r: 0.020 | r: 0.044 | r: 0.038 | r: −0.038 | r: −0.010 |
| Carbohydrate (E%) | | r: 0.021 | r: −0.003 | r: 0.062 | r: 0.007 | r: -0.070 | r: −0.010 | r: 0.060 |
|  | Dietary fibre (g/1000 kcal) | r: −0.044 | r: 0.007 | r: −0.005 | r: 0.030 | r: 0.032 | r: 0.070 | r: −0.003 |
| Fat (E%) | | r: 0.260 | r: 0.060 | r: 0.021 | r: −0.485 | r: 0.057 | r: 0.030 | r: −0.070 |
| Protein (E%) | | r: −0.028 | r: -0.042 | r: −0.102* | r: 0.006 | r: -0.031 | r: −0.009 | r: −0.062 |
| ***Food group (g/1000 kcal)*** | |  |  |  |  |  |  |  |
| Corn | | r: -0.001 | r: 0.002 | r: 0.010 | r: 0.080* | r: 0.001 | r: 0.009 | r: −0.006 |
| Vegetables | | r: 0.003 | r: 0.002 | r: −0.034 | r: −0.009 | r: 0.060 | r: 0.021 | r: −0.028 |
| Fruit and berries | | r: −0.022 | r: 0.016 | r: 0.022 | r: 0.045 | r: 0.062 | r: 0.008 | r: 0.020 |
| Meat | | r: -0.010 | r: 0.057 | r: 0.010 | r: 0.027 | r: 0.010 | r: −0.031 | r: −0.020 |
| Fish | | r: −0.066 | r: −0.040 | r: −0.068 | r: 0.022 | r: 0.021 | r: −0.023 | r: 0.036 |
| Egg | | r: 0.033 | r: 0.039 | r: 0.023 | r: 0.039 | r: 0.074 | r: −0.042 | r: −0.043 |
| Dairy products | | r: 0.036 | r: −0.017 | r: 0.030 | r: 0.010 | r: −0.010 | r: 0.001 | r: −0.127* |
| Butter/Oil | | r: 0.041 | r: 0.070 | r: 0.074 | r: 0.048 | r: 0.065 | r: −0.008 | r: 0.039 |

Asterisk (***)** indicates statistical significance (p<0.05). BSS, Bristol Stool Scale; IBS, Irritable Bowel Syndrome; GSRS, Gastrointestinal Symptom Rating Scale; IBS-SSS, Irritable Bowel Syndrome – Severity Scoring System.
